# Anthelmintic activity of winter savory (*Satureja montana* L.) essential oil against gastrointestinal nematodes of sheep

**DOI:** 10.1186/s12917-025-04771-3

**Published:** 2025-06-07

**Authors:** Filip Štrbac, Slobodan Krnjajić, Radomir Ratajac, Laura Rinaldi, Vincenzo Musella, Fabio Castagna, Dragica Stojanović, Nataša Simin, Dejan Orčić, Antonio Bosco

**Affiliations:** 1 Institute for Multidisciplinary Research, University of Belgrade, Belgrade, Serbia; 2 Scientific Veterinary Institute Novi Sad, Novi Sad, Serbia; 3 Department of Veterinary Medicine and Animal Production, University of Naples Federico II, CREMOPAR, Naples, Italy; 4 Department of Health Sciences, University of Catanzaro Magna Græcia, Catanzaro, Italy; 5 Department of Veterinary Medicine, Faculty of Agriculture, University of Novi Sad, Novi Sad, Serbia; 6 Department of Chemistry, Biochemistry and Environmental Protection, Faculty of Sciences, University of Novi Sad, Novi Sad, Serbia

**Keywords:** Anthelmintic resistance, Anthelmintic drug residues, Botanical anthelmintics, Integrated parasite control, In vitro test, In vivo test, Toxicity study, Chemical analyses, Coproculture

## Abstract

**Background:**

The increasing difficulties in combating anthelmintic resistance in gastrointestinal nematodes (GINs) of sheep worldwide, and the residues of chemical drugs in animal products and the environment, necessitate the search for alternatives. Previous studies have shown that plant essential oils (EOs) could be valuable anthelmintic agents, due to their numerous advantages. The present study aimed to evaluate the possibility of using winter savory (*Satureja montana* L.) EO against sheep GINs. The chemical composition of the tested oil was determined by gas chromatography-mass spectrometry (GC–MS). The efficacy of the tested oil was determined in vitro using the egg hatch test (EHT), and in vivo using the faecal egg count reduction test (FECRT) performed in two farms. Preliminary toxicity studies including clinical observation, haematological and biochemical blood analysis were also performed to evaluate the safety of the tested oil to the hosts. In addition, a coproculture study was carried out in the tested farms using the appropriate morphological keys.

**Results:**

Main compounds of the *S. montana* oil identified by GC–MS analysis were *p*-cymene (42.8%), carvacrol (28.1%) and y-terpinene (14.6%). The in vitro EHT showed a dose-dependent (R^2^ = 0.94) anthelmintic potential of the tested oil, with ovicidal activity varying from 17.0–83.3% and determined IC_50_ value of 0.59 mg/ml. The field efficacy reached 33% (at group level) and 50% (at individual level) at D14 after treatment. In vivo efficacy was significantly higher in farm 2 (FEC above 65% at group level, *p* < 0.05) where sheep were kept in pens during treatment. No toxic effects were observed, either in the physical observation of the test animals or in their liver and kidney function. No significant changes (*p* > 0.05) in the percentage representation of GIN genera were observed in the coproculture study, indicating that the treatment agent was not specific to a single genus.

**Conclusion:**

The anthelmintic potential showed on EHT and FECRT, without adverse effects on the sheep, suggests that *S. montana* EO is suitable for the control of sheep GINs as part of an integrated parasite management. However, further studies should be conducted to increase efficacy in field conditions.

## Introduction

As a valuable source of meat, milk (and its products), wool and manure, sheep represent an important sector of the livestock industry worldwide [1]. This is especially true given the increasing demand for food for the growing human population [2]. However, modern sheep farming is facing an increasing problem of infections caused by gastrointestinal nematodes (GINs), which are consider as a major obstacle to sustainable sheep production from both a health and economic perspective [3–5]. Depending on the level of worm burden, these parasites can have various negative effects on sheep. These include reduced appetite and feed intake, weight loss, anaemia, diarrhoea and hypoproteinemia resulting in reduced productivity, immunity and fertility, even death in cases of high parasite infection [1, 6]. All of this can lead to enormous economic losses, estimated at hundreds of millions of euros per year [7]. In addition, the occurrence of GIN infections is predicted to increase greenhouse gas emissions by up to 30% [8, 9].

Therefore, it is clear that the issue of controlling these parasites is a top priority, which requires the development of sustainable strategies. To date, heavy reliance has been placed on the use of commercial anthelmintics from different chemical classes (benzimidasoles, macrocyclic lactones and imidazothiazoles) in an attempt to maintain the number of parasites below levels that can cause disease, whether clinical or subclinical [9]. However, the extensive and inappropriate use of these drugs has led to the emergence and development of anthelmintic resistance (AR) in nematodes to all major groups of drugs, sometimes simultaneously to several different classes [10, 11]. Annual costs due to AR development in Europe alone are estimated at €38 million and rising, with underdosing, overfrequent treatments, mass treatment, and single-drug regimens being the main risk factors [9, 12]. In addition, the exclusive use of chemical drugs is associated with the occurrence of residues in animal products and in the environment, leading to growing concerns about food safety and public health [11, 13–15]. All of these justify the search for alternative solutions and their utilization in the future management of GIN infections.

Medicinal plants have been used worldwide for thousands of years as a valuable source for the treatment of various diseases in humans and animals [15]. Although these practices have been replaced by modern medicine, interest in phytotherapy has increased recently for many reasons. They are generally considered to be effective while being safer and cheaper than chemical medicines [16]. For these reasons, herbal medicines may be a suitable option (especially for the treatments in organic livestock) and generally help to reduce the use of commercial antibiotics and antiparasitics [16–18]. EOs represent plant secondary metabolites, i.e. volatile, aromatic and viscous components obtained from different part of plants through well-regulated extraction processes [19, 20]. Therefore, they contain various bioactive substances with great pharmacological potential that can be exploited for various indications in veterinary medicine [18, 21–23]. Moreover, EOs from various plants have already been investigated for their anthelmintic activity against GINs in sheep, with some of them showing promising results [24, 25].

In this context, winter or mountain savory (*Satureja montana* L. sensu lato) is a perennial, aromatic wild shrub that grows along or near the Adriatic coast, but also in the Pyrenees [26]. It is reported in many countries in this region including Serbia [27] and Italy [28]. *S. montana* is a well-known medicinal plant with a high biological potential that has been used in traditional medicine. Thus, its antimicrobial, antioxidant, digestive, antidiuretic and other properties have been used for the traditional treatment of various ailments such as infectious diseases, diarrhea, muscle pain, nausea, etc. [29, 30]. In addition, winter savory is used in the food industry as a spice and flavoring agent [26]. However, the anthelmintic potential of this plant against gastrointestinal nematodes in sheep has not yet been fully explored. Therefore, the aim of this study was to demonstrate the possibility of using *S. montana* EO against GINs in sheep. The hypothesis is that this EO has anthelmintic potential against these parasites, which can be demonstrated by regular in vitro and in vivo tests including the egg hatch test and the faecal egg count reduction test, while at the same time it is safe for the sheep.

## Material and methods

### Chemical composition

The essential oil of *Satureja montana* (L.) was obtained from the Institute of Field and Vegetable Crops, Novi Sad, Serbia. The steam distillation of the fresh aerial plant parts of *S. montana* L. was performed in a semi-industrial distillation unit according to the procedure described by Aćimović et al. [31]. The chemical composition (qualitative and semi-quantitative characterization) of the tested oil was determined by gas chromatography-mass spectrometry (GC–MS) at the Department of Chemistry, Biochemistry and Environmental Protection, Faculty of Sciences, University of Novi Sad, Serbia. Analyses were performed using an Agilent Technologies gas chromatograph coupled with an Agilent Technologies 5975B electron ionization mass-selective detector using the technical conditions as described by Knežević et al. [32]. Data were acquired in scan mode (m/z range 35–400), with a solvent delay of 2.30 min, and processed using Agilent Technologies MSD ChemStation software (revision E01.01.335) combined with AMDIS (ver. 2.64) and NIST MS Search (ver. 2.0 d). Compounds were identified by comparison of mass spectra with data libraries (Wiley Registry of Mass Spectral Data, 7 th ed., and NIST/EPA/NIH Mass Spectral Library 05) and confirmed by comparison of linear retention indices with literature data [33]. The relative amount of each component is expressed as a percentage of its peak area relative to the total peak area.

### Experimental animals

The remaining analyses were carried out at the Regional Center for Monitoring Parasitic Infections (CREMOPAR, Campania region, southern Italy). Two farms in this region with a previously identified high prevalence of GINs, in an area with a typical Mediterranean climate, were used for the trials, where the animals were kept in free range (Farm 1) and in boxes (Farm 2) during the treatment.

In this region, extensive farming is still very widespread. With 6,707 sheep farms and 160,784 sheep, on a national scale, the region occupies the six positions for the number of farms and the seven position for the number of sheep. These data, updated as of 30 June 2024 were provided by the National Database (BDN) of the Zootechnical Registry-CSN of the “G. Caporale” Institute of Teramo (Italy). The two farms, already monitored by the CREMOPAR research group, have never shown any signs of drug resistance so far.

Thus, sheep with natural mixed infection, mainly a mixture of Lacaune and Bagnolese dairy breeds, of similar age (2 ± 0.5 years), grazing season and body weight (b.w.) of 50 ± 5 kg were used for the present study. However, animals with different faecal egg counts (up to 3640 EPG) were selected to simulate natural conditions. The animals selected for the study were all lactating females. They were fed with pasture and forage (barley and maize grains) with no changes during the trial. The hilly pasture (average altitude 390 m above sea level) is made up of spontaneous forage plants typical of the Mediterranean area, and the animals graze all together throughout the year regardless of sex and age. All the animals had not received anthelmintic treatments in the past six months. The last treatment was performed eight months before the trial with oral albendazole. The animals were fasted before and 2 h after the application of the treatments, as is usual when using commercial anthelmintics.

### In vitro egg hatch test

Anthelmintic potential in vitro of *S. montana* EO was evaluated using the egg hatch test (EHT), with the recovery method described by Bosco et al. [6, 34] was used to obtain GIN eggs. The faecal samples used for the in vitro tests came from the two sheep farms used for the in vivo tests. For this purpose, faecal samples (*n* = 30) were collected directly from the rectal ampulla of randomly selected sheep, transported to the laboratory at a temperature of 10 °C and processed within 2 h of collection. To isolate GIN eggs, faecal samples were pooled, homogenised and then filtered under running water through meshes of 1 mm, 250 μm, 212 μm and 38 μm size. Subsequently, the eggs retained at the last size were washed with distilled water and centrifuged at 52.36 rad/s, and the supernatant was discarded. The eggs were then floated by centrifugation with 40% sugar solution, isolated into new tubes and mixed with distilled water. Two more centrifugations were performed to remove the pellets, after which an aqueous solution containing GIN eggs was obtained.

As in our previous studies [35, 36], the EHT was performed at eight different concentrations (50, 12.5, 3.125, 0.781, 0.195, 0.049, 0.025 and 0.0125 mg/mL) of the tested oil. Twenty-four well plates were used for the experiments, whereby different oil concentrations were emulsified in Tween 80 (3%, v/v) and added to wells containing aqueous solutions (40 μL) of approximately 150 eggs/well. The positive control was thiabendazole (TBZ, Sigma, Saint Louis, MO, USA) at the two lowest concentrations used for EO, and the negative controls were emulsifier and distilled water. All wells were completed with distilled water to obtain a final volume of 0.5 mL/well, and incubated at a constant temperature of 27 °C for 48 h. The incubation was stopped with Lugol's solution, after which the eggs and first-stage larvae (L1) were counted under an inverted microscope. The experiment was performed in three replicates, and the values obtained were expressed as the arithmetic mean of the inhibition of egg hatchability for each concentration.

### In vivo faecal egg count reduction test

The field trial was conducted using the faecal egg count reduction test (FECRT) on two different farms. In each of them, the animals (*n* = 36/farm, 72 in total) were divided into three groups which were treated as follows:

- G1: *Satureja montana* EO, 150 mg/kg (*n* = 12).
- G2: Albendazole, 3.8 mg/kg (*n* = 12), positive control.
- G3: Sunflower oil, 50 mL (*n* = 12), negative control.

To further describe the natural formulation used for this test, *S. montana* EO was mixed with sunflower oil in a ratio 1:4.5 to avoid the effect of the pure EO on the gastrointestinal mucosa. The quantity of the EO given to each tested animal was expressed in a given mg/kg of body weight, and the final volume of the formulation (EO + suflower oil) was 50 mL per animal. All treatments were administered once (single dose), directly into the rumen of the animals using a tube inserted through the oral cavity, pharynx and oesophagus (intraruminal application). For each group, a single tube was used and rinsed from one animal to another. Individual faecal samples were collected rectally at Day 0, D7 and D14 after treatments and stored at 4 °C before processing. For counting of eggs, novel Mini-FLOTAC technique [37] was used with a detection limit of 5 eggs per gram (EPG) of faeces, and using a sodium chloride flotation solution (specific gravity = 1.200). The farms were chosen based on previously determined occurrences of natural-mixed GIN infections. On both farms, animals for the trial were randomly selected for each group and thus were different in worm burden to completely simulate the conditions in the practice.

### Clinical observation, haematological and biochemical blood analyses

All treated animals were observed clinically at the sampling times (D0, D7 and D14) for the presence of adverse effects of the administered EO, with particular attention paid to their feed, defecation, and behaviour. In addition, blood samples were taken from randomly selected animals from each group (two farms, *n* = 6/group, 36 in total) at D0 and D14 to assess the effects of the EO on blood parameters. For haematological parameters, samples were placed in EDTA-containing vacuum tubes and processed within 2–4 h. This evaluation reflected the potential presence of toxic effects of the applied EO, but also its impact on reducing signs of anaemia caused by blood-sucking nematodes. The reference values were used according to the Sajid et al. [38] with some modifications. In contrast, blood samples were collected for biochemical analyses in empty vacuum tubes and subsequently analysed, and these analyses reflected the effects of EO on the kidney (urea, creatinine) and liver (aspartate aminotransferase, AST, and gamma-glutamyl transferase, GGT) function.

### Coproculture examination

For the identification of GIN genera present on the tested farms, coproculture examination was performed following the protocol developed by the UK Ministry of Agriculture, Fisheries and Food [39]. For each of the groups for the in vivo test (*S. montana* EO, albendazole and sunflower oil), an equal amount of faeces was collected to form a pool for coproculture study. These were performed for each of the sampling time points (D0, D7 and D14) to evaluate the effects of the treatments on the percentage representation of the nematode genera. Third-stage larvae (L3) developed were identified using the morphological determination keys proposed by van Wyk and Mayhew [40]. The identification and percentages of each nematode genera were performed on 100 L3, identifying all larvae if the sample contained 100 or fewer L3. In this way, it was possible to determine the percentage of each GIN genus in the total number of identified larvae.

### Statistical analyses

The inhibitions of egg hatchability (IH) in the in vitro test were calculated using the following formula [41, 42]:

$$\text{IH }({\%}) = [(\text{number of eggs})/(\text{number of larvae }+\text{ number of eggs})] \times 100$$

To evaluate the significance of the differences (*p* < 0.05) between the values obtained for different EO concentrations with each other and with controls, a one-way analysis of variance (ANOVA) with post-hock Tuckey`s test was performed. In addition, a nonlinear regression/logarithmic distribution was applied to determine the half-maximal inhibitory concentration (IC_50_) and to evaluate the presence of a dose-dependent effect [43].

The reductions in the faecal egg counts in the in vivo test were calculated using the following formula [41, 44]:

$$\text{FECR }({\%})=\hspace{0.17em}[1\hspace{0.17em}-\hspace{0.17em}(\text{T}2/\text{T}1\hspace{0.17em}\times \hspace{0.17em}\text{C}1/\text{C}2)]\hspace{0.17em}\times \hspace{0.17em}100$$

In this formula, T_1_ and T_2_ represent the average EPGs (arithmetic means) before (D0) and after treatment (D7 or D14) in the *S. montana* EO or albendazole group, while C_1_ and C_2_ represent the average EPGs before (D0) and after treatment (D7 or D14) in the group that received sunflower oil. Results were analysed using a two-way ANOVA followed by Tukey`s test to evaluate the presence of significant differences (*p* < 0.05) in EPG values obtained within one group and farm on different time points, to compare EPGs in different groups at the same time point and farm, but also to compare EPGs between two farms. The final results represent the mean value from both farms.

The results of the haematological and biochemical blood analyses were also analysed using two-way ANOVA. The post-hock Sidak`s test (*p* < 0.05) was used to compare the values of individual parameters in the same group on D0 and D14. In contrast, for the comparison of the values of individual parameters obtained in different groups on the same day, the post-hock Tukey’s test was performed (*p* < 0.05). Finally, the same tests were performed in the analysis of the results of the coproculture examination to evaluate the differences in the percentage of each GIN genera before and after the treatment in each group. 

All statistical analyses were performed using GraphPad Prism 10.1.2. (GraphPad Holdings, LLC, San Antonio, CA, USA).

## Results

### Chemical composition

**Fig. 1:**
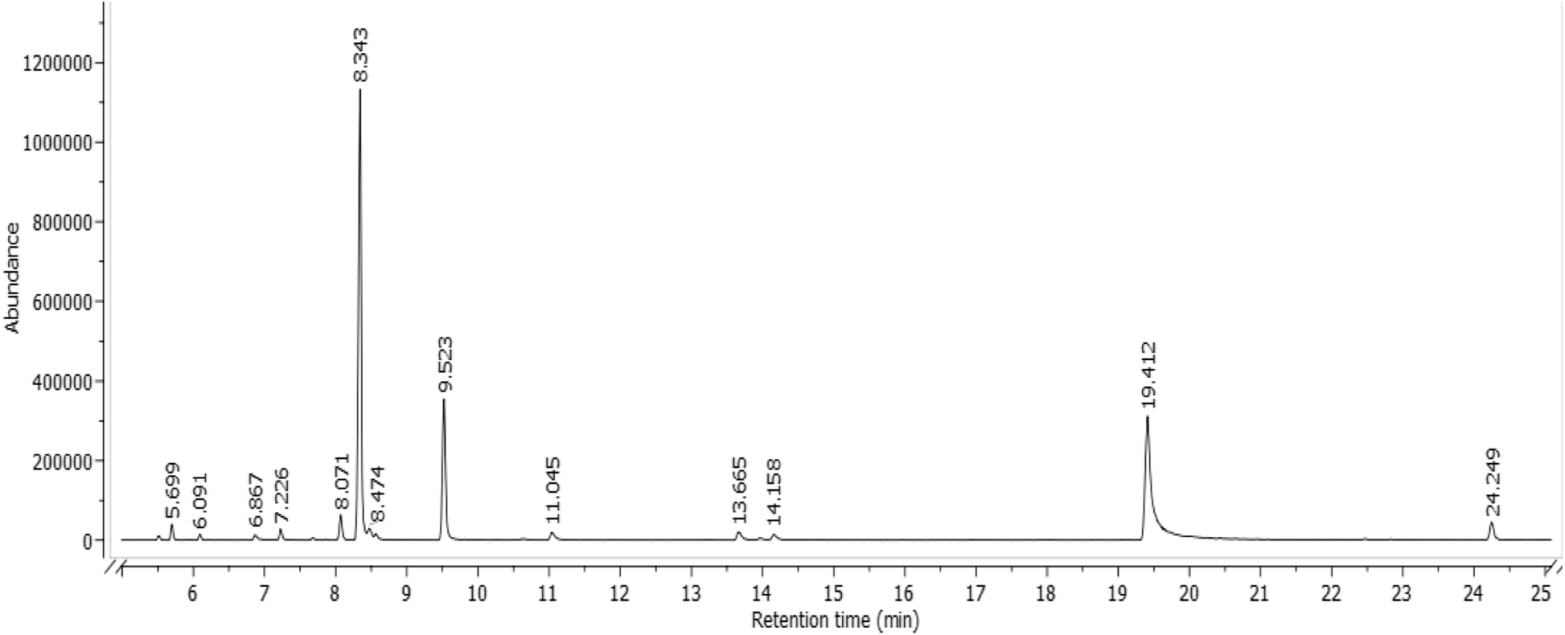
GC–MS chromatogram of the *Satureja montana* essential oil

A total of 17 compounds belonging to different chemical groups were identified by GC–MS analysis (Table 1, Figure 1). The most abundant group of compounds were hydrocarbon monoterpenes, i.e. p-cymene (42.8%), γ-terpinene (14.6%), β-caryophyllene (2.46%), α-terpinene (2.32%), limonene (1.52%) and α-pinene (1.16%). However, the a phenolic terpenoid carvacrol (28.1%) and the alcoholic terpenoids borneol (1.27%) and linalool (1.20%) were also represented. The rest of the compounds were present in less than 2%.

**Table 1:** Chemical composition (% of total peak area) of the *Satureja montana* (L.) essential oil identified by gas chromatography–mass spectrometry analysis

AI	Compound	% of total peak area
925	α-Thujene	0.33
932	α-Pinene	**1.16**^**a**^
947	Camphene	0.44
976	β-Pinene	0.66
990	β-Myrcene	0.96
1005	α-Phellandrene	0.18
1016	α-Terpinene	**2.32**
1024	p-Cymene	**42.8**
1027	Limonene	**1.52**
1030	1,8-Cineole	0.73
1057	γ-Terpinene	**14.6**
1088	α-Terpinolene	0.17
1100	Linalool	**1.20**
1164	Borneol	**1.27**
1176	Terpinen-4-ol	0.78
1302	Carvacrol	**28.1**
1418	β-caryophyllene	**2.46**
Total % of identified compounds		**99.7**

^*^AI—arithmetic retention index; ^a^—compounds with abundance > 1% are written in bold

### In vitro egg hatch test

In the EHT, *S. montana* EO showed ovicidal activity with an inhibition of egg hatchability that varied from 17.3 to 83.0%, depending on the concentration used (Table 2). The effect was dose-dependent (R^2^ = 0.94) with a determined IC_50_ value of 0.59 mg/mL. At all of the concentrations tested, the activity of the tested EO was significantly higher than that of the two negative controls (*p* < 0.05).

**Table 2:** The inhibitory effect (mean ± standard deviation) of different concentrations of the *Satureja montana* L.) essential oil on egg hatching of gastrointestinal nematodes in sheep

Concentration of EO [mg/mL]	Inhibition of hatchability (%)
50	83.0 ± 2.00^B^
12.5	70.0 ± 2.65^C^
3.125	61.0 ± 2.00^D^
0.781	56.7 ± 1.53^D^
0.195	48.0 ± 1.00^E^
0.049	22.0 ± 1.00^F^
0.025	20.0 ± 1.00^F^
0.0125	17.3 ± 2.52^F^
Control (+)^1^	96.3 ± 1.53^A^
Control (+)^2^	95.0 ± 1.00^A^
Control (−)^1^	8.0 ± 1.00^G^
Control (−)^2^	6.60 ± 1.92^G^

^*^Uppercase compares means between different concentrations and controls. Different letters indicate significant differences (*p* < 0.05). Control (+)^1^—thiabendazole, 0.025 mg/mL; control (+)^2^—thiabendazole, 0.0125 mg/mL; control (−)^1^—3% Tween 80, v/v; control (−)^2^—distilled water; EO—essential oil

### In vivo faecal egg count reduction test

**Fig. 2:**
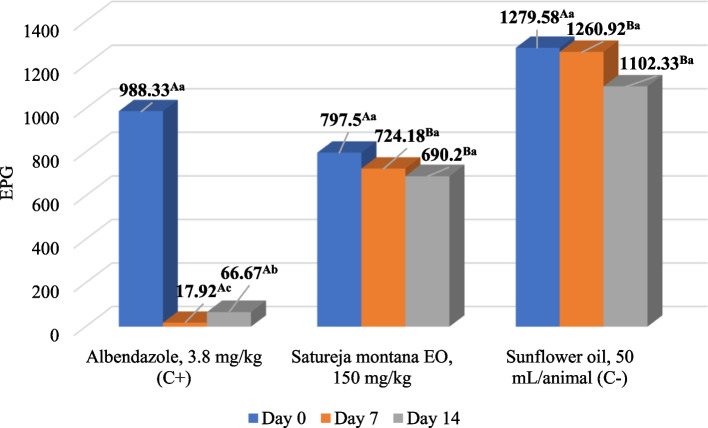
Mean reduction of eggs per gram (EPG) of faeces of gastrointestinal nematodes in sheep treated with *Satureja montana* EO, albendazole and sunflower oil on Farm 1. Uppercase compares means between different groups at one time point; lowercase compares means of different time points within the group. Different letters indicate significant differences (*p* < 0.05); EPG - eggs per gram; EO – essential oil; C – control

**Fig. 3:**
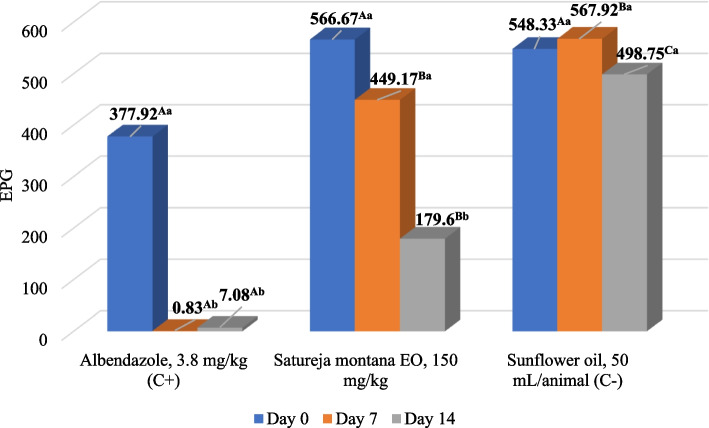
Mean reduction of eggs per gram (EPG) of faeces of gastrointestinal nematodes in sheep treated with *Satureja montana* EO, albendazole and sunflower oil on Farm 2. Uppercase compares means between different groups at one time point; lowercase compares means of different time points within the group. Different letters indicate significant differences (*p* < 0.05). EPG – eggs per gram; C – control

In the field trial, *S. montana* EO showed anthelmintic activity with an overal average group EPG reduction of 15.7% and 33.0% in total at D7 and D14 post-treatment, respectively (Table 3). In contrast to the low activity on Farm 1 (Figure 2), the effect was significantly higher on Farm 2 (*p* < 0.05), where the EPG reduction at group level reached 68.3% at D14 after treatment. Also, the average EPG value at this time point was significantly lower when compared to the pre-treatment value, and the effect was significantly higher than the negative control on that Farm (*p* < 0.05) (Figure 3). However, there were no significant differences between the EPG values from the treated group and the negative control in total (*p* > 0.05). If each animal is observed separately, the median individual reduction was higher than the arithmetic average of the group level and reached 18.9% and 50.0% in total on D7 and D14 post-treatment, respectively. In the albendazole-treated group, there was significant total reduction of EPG (*p* < 0.05)(Table 3).

**Table 3:** Average (arithmetic mean ± standard deviation) reduction of eggs per gram of gastrointestinal nematodes in sheep treated with *Satureja montana* essential oil – in total from both examined farms

Treatment		Day 0	Day 7	Day 14
*S. montana* EO, 150 mg/kg	EPG	682.1 ± 632.9.^Aa^	586.7 ± 552.9^Aa^	434.9 ± 404.0^Aa^
	FECR_(group)_	/	15.7%	33.0%
Albendazole, 3.8 mg/kg (Control +)	EPG	683.1 ± 735.5^Aa^	9.38 ± 11.8^Bb^	36.9 ± 41.6^Bc^
	FECR_(group)_	/	99.0%	95.1%
Sunflower oil, 50 ml (Control -)	EPG	914.0 ± 821.9^Aa^	914.4 ± 784.8^Aa^	800.5 ± 680.3^Aa^

^*^ Uppercase compares means between different groups at one time point; lowercase compares means of different time points within the group. Different letters indicate significant differences (*p* < 0.05); EPG—eggs per gram; EO – essential oil; FECR – faecal egg count reduction

### Clinical observation, haematological and biochemical blood analyses

No side effects were observed during clinical observation of the examined animals. The haematological analyses showed that there were no significant changes in the values of the tested parameters after any of the applications (*p* > 0.05). Moreover, the applied *S. montana* EO, as well as albendazole, slightly increasing the levels of RBC, Hgb and HCT, while at the same time those levels decreased in the negative control group (Table 4). In the biochemical analyses, no significant increase was observed in any of the parameters tested (*p* > 0.05). In fact, the mean value of GGT at D14 was significantly lower than the pre-treatment value (*p* < 0.05) (Table 5).

**Table 4:** Effect (mean ± standard deviation) of the application of *Satureja montana* EO on haematological blood parameters

Parameter	Reference values	Day	*Satureja montana* EO	Albendazole, C (+)	Sunflower oil, C (-)
WBC (K/uL)	4.0–12.0	0	9.61 ± 2.0	9.59 ± 1.6	8.78 ± 2.9
		14	9.95 ± 2.5	9.45 ± 2.6	8.74 ± 2.3
RBC (M/uL)	8.0–16.0	0	7.67 ± 1.4	7.50 ± 0.5	8.01 ± 1.0
		14	7.74 ± 0.9	7.66 ± 0.6	7.45 ± 1.1
Hgb (g/dL)	8.0–16.0	0	10.8 ± 1.3	10.4 ± 0.5	11.7 ± 1.8
		14	11.4 ± 0.9	11.1 ± 0.8	11.0 ± 1.7
HCT (%)	24.0–50.0	0	27.6 ± 3.3	26.4 ± 1.6	29.4 ± 4.7
		14	28.0 ± 1.9	27.3 ± 1.8	27.4 ± 4.2
MCV (fL)	23.0–48.0	0	36.4 ± 3.9	35.2 ± 1.7	36.8 ± 2.4
		14	36.5 ± 3.0	35.7 ± 2.0	36.8 ± 1.8
MCH (Pg)	9.0–12.0	0	14.3 ± 1.4	13.9 ± 0.6	14.6 ± 0.8
		14	14.9 ± 1.1	14.5 ± 0.7	14.7 ± 0.8
MCHC (g/dL)	31.0–38.0	0	39.2 ± 0.9	39.5 ± 1.0	39.7 ± 0.8
		14	40.8 ± 0.8	40.5 ± 0.7	40.1 ± 0.9
RDW (%)	-	0	16.0 ± 1.0	15.5 ± 0.5	15.9 ± 0.7
		14	16.2 ± 0.9	15.8 ± 0.5	15.7 ± 0.8
Pit (K/uL)	250—750	0	567.3 ± 215.6	517.0 ± 119.0	545.1 ± 189.1
		14	522.2 ± 179.9	468.2 ± 142.0	483.0 ± 207.7
MPV (fL)	-	0	11.5 ± 2.1	11.3 ± 1.5	11.1 ± 1.3
		14	11.5 ± 1.9	11.4 ± 1.6	10.8 ± 1.3
Pct (%)	-	0	0.57 ± 0.2	0.60 ± 0.6	0.58 ± 0.2
		14	0.61 ± 0.3	0.54 ± 0.2	0.46 ± 0.1
PDW (%)	-	0	6.22 ± 8.4	5.43 ± 4.5	5.83 ± 8.0
		14	9.51 ± 9.50	4.59 ± 5.0	9.23 ± 9.6
Lin (K/uL)	-	0	5.73 ± 1.3	4.94 ± 1.3	4.16 ± 1.1
		14	5.97 ± 1.9	5.41 ± 2.5	4.73 ± 1.6
Lin (%)	40—75%	0	59.5 ± 6.9	51.8 ± 12.0	52.8 ± 12.3
		14	60.3 ± 1.9	56.4 ± 17.1	54.2 ± 6.8
Gra (K/uL)	-	0	3.9 ± 1.0	4.65 ± 1.4	4.09 ± 2.4
		14	4.0 ± 1.3	4.04 ± 1.7	4.01 ± 1.2
Gra (%)	-	0	40.5 ± 6.9	48.2 ± 12.0	47.2 ± 12.3
		14	39.7 ± 10.8	43.6 ± 17.1	45.8 ± 6.8

^*^ No statistically significant differences were found when comparing values on D14 compared to D0, nor when comparing values for different groups on the same day (*p* > 0.05)

**Table 5:** Effect (mean ± standard deviation) of the application of *Satureja montana* essential oil on the biochemical blood parameters reflecting renal and hepatic function in the tested animals

Treatment	Day	Urea (mg/dL)	Creatinine (mg/dL)	AST (UI/L)	GGT (UI/L)
*S. montana* EO	0	31.0 ± 8.74^Aa^	14.1 ± 3.03^Aa^	180.3 ± 61.10^Aa^	71.8 ± 7.62^Aa^
	14	30.8 ± 6.98^Aa^	11.8 ± 3.41^Aa^	157.8 ± 42.10^Aa^	65.0 ± 3.77^Bb^
Albendazole (Control +)	0	27.6 ± 9.45^Aa^	12.4 ± 2.75^Aa^	173.3 ± 82.72^Aa^	72.0 ± 8.86^Aa^
	14	28.4 ± 6.07^Aa^	13.4 ± 3.68^Aa^	163.0 ± 50.72^Aa^	66.5 ± 5.54^Bab^
Sunflower oil (Control -)	0	29.8 ± 7.87^Aa^	12.4 ± 2.84^Aa^	189.7 ± 72.63^Aa^	72.5 ± 7.82^Aa^
	14	29.3 ± 4.91^Aa^	12.8 ± 3.04^Aa^	206.9 ± 56.32^Aa^	73.7 ± 5.30^Aa^

^*^ Uppercase compares means between values at different time points within one treatment group and lowercase values at the same time points between different treatment groups. Different letters indicate significant differences (*p* < 0.05); AST - aspartate aminotransferase, GGT - gamma-glutamyl transferase, EO - essential oil

### Coproculture examination

Four genera of sheep GINs were identified on both tested farms during the coproculture examination. In total, they were represented on D0 as follows: *Haemonchus* 45%, *Trichostrongylus* 32%, *Teladorsagia* 19% and *Chabertia* 4%. After treatment, the percentages changed slightly, but with no difference between the groups (*p* > 0.05). Similarly, changes observed before and after treatment were not statistically significant in any of the tested groups (*p* > 0.05). The percentages of each GIN genera in each in vivo treatment group in both farms before (D0) and after treatment (D7 and D14) are shown in Tables 6 and 7.

**Table 6:** Distribution of sheep nematode third-stage larvae (L3) genera (%) at different time points (D0, D7 and D14) on Farm 1

Day	Group	*Haemonchus*	*Trichostrongylus*	*Teladorsagia*	*Chabertia*	Total
0	*Satureja montana* EO	51	22	23	4	100
	Albendazole (Control +)	42	26	28	4	100
	Sunflower oil (Control -)	48	21	30	1	100
7	*Satureja montana* EO	53	26	18	3	100
	Albendazole (Control +)	56	21	15	8	100
	Sunflower oil (Control -)	46	29	22	3	100
14	*Satureja montana* EO	52	23	17	8	100
	Albendazole (Control +)	44	30	21	5	100
	Sunflower oil (Control -)	49	25	19	7	100

^*^ No statistically significant differences were found when comparing the values of different treatments within one time point, nor comparing the values obtained at different time points within one treatment (*p* > 0.05); EO – essential oil

**Table 7:** Distribution of sheep nematode third-stage larvae (L3) genera at different time points (D0, D7 and D14) on Farm 2

Day	Group	*Haemonchus*	*Trichostrongylus*	*Teladorsagia*	*Chabertia*	Total
0	*Satureja montana* EO	41	45	11	3	100
	Albendazole (Control +)	38	42	19	1	100
	Sunflower oil (Control -)	43	35	20	2	100
7	*Satureja montana* EO	39	43	15	3	100
	Albendazole (Control +)	42	38	20	0	100
	Sunflower oil (Control -)	38	41	16	5	100
14	*Satureja montana* EO	37	45	13	5	100
	Albendazole (Control +)	41	39	19	1	100
	Sunflower oil (Control -)	37	43	18	2	100

^*^No statistically significant differences were found when comparing the values of different treatments within one time point, nor comparing the values obtained at different time points within one treatment (*p* > 0.05); EO – essential oil

## Discussion

The search for new anthelmintics requires the use of appropriate methods to test their efficacy. From this point of view, the EHT is used for studying the ovicidal activity of drugs and it is suitable for an initial assessment of anthelmintic potential and forms a basis for other tests. Although the larval development test (LDT) has an advantage due to its higher sensitivity [45], the EHT is less labour-intensive and requires less time to perform [46], with both tests showing comparable and reliable results and having an advantage over other in vitro tests. For this reason, it is the most commonly used in vitro test for the evaluation of the anthelmintic activity of drugs, i.e. benzimidazoles, and for the detection of resistance [47]. At the same time, it is commonly used for the evaluation of the anthelmintic potential of EOs [25]. In the EHT performed in the present study (Table 2), the EO of *S. montana* showed ovicidal activity varying from 17.3–83.0%, with an IC_50_ value of 0.59 mg/mL. Although the maximum inhibitory effect (100%) on hatching of GINs eggs was observed in our previous study [48], and the obtained efficacy of 83.0% at the very high concentration of 50 mg/mL can be consieder as low, the IC_50_ value obtained in the present study is still high compared to oils from other studies [24, 25], indicating its anthelmintic potential. Indeed, this parameter is considered suitable for comparing the drug-inhibitory effect of different active substances [49].

The results obtained for two samples of *S. montana* EO in the present and the previous study [48] indicate a high variability in the efficacy of EOs, since both samples were obtained from the same producer and had the same chemical composition. However, in addition to the known factors that can influence the presence and abundance of the compounds (geographical origin of the plant, light, rainfall, soil conditions, age and part of the plant, genetic characteristics, presence of certain organisms and microorganisms, etc.) and thus their effect, many other factors related in post-extraction period may also be involved. That esspecially refers to the way the EOs are stored and the length of time before they are used, as they are sensitive to light, temperature and oxygen [50, 51]. However, not only the chemical composition of the EOs can influence their effect against GINs. Immunity of the animals is generally important for the host – GINs parasitism interaction [52] and thus factors such as genetic constitution, age and physiological status of the animals, management factors inlcuding nutrition, etc. are also important for the sucessfull treatment and positive outcome.

 On the other hand, in vivo FECRT is considered the method of choice for evaluating the field efficacy of anthelmintic compounds and the detection of anthelmintic resistance, as it is reliable, practical and is intended for all anthelmintic classes across all animal species. Furthermore, it can be performed for multiple parasitic species and do not require sacrificing the animals [47, 53]. Although even more reliable, the controlled efficacy test (CET) is not practicable in the field as it is it is labour-intensive, time-consuming and costly (animals are artificially infected, treated, then slaughtered and worm burden counted) [54]. For these reasons, FECRT is the most commonly used method for evaluating the anthelmintic efficacy of drugs in practice [55]. Examination of the effect of *S. montana* EO in vivo was performed for the first time to our knowledge, and it showed limited activity with a total reduction of 33.0% on D14 after treatment (Table 3). While in Farm 1 the applied oil was less effective, in Farm 2 it significantly reduced the EPG on D14 after treatment and reached an efficacy of 68.3% (Figure 3). A similar trend was also observed in our previous studies [35, 36] conducted with oregano and mint EOs, where these oils were also much more effective in Farm 2. This can be explained by the differences in animal husbandry (free range in comparison with the boxes), which affect the manipulation of the animals during treatment, but may also have an influence on the gastrointestinal tract of animals in general due to differences in their feeding. However, the average individual median reduction was higher than that at the group level, reaching 50% in total on D14, suggesting that EO had an effect in most of the tested animals.

The lower efficacy in vivo of EOs in general can be attributed to their unstable nature as their ingredients are prone to degradation and evaporation, which causes limited bioavailability [51, 56]. On the other hand, the complexity of the gastrointestinal tract of ruminants that hinders oral applications is also involved [57]. For these reasons, the EO was administered in a specific, inovative way in the present study – intraruminally via a tube placed through the oral cavity, pharynx and oesophagus. This method of administration not only avoids the possible effect of the formulation on the mucous membranes of the upper parts of the gastrointestinal tract, but also the inactivation of the EO active ingredients in these parts. It also better ensures that the exact amount of EO can reach the target sites in the abomasum and intestine compared to peroral administration or administration via the feed, where a certain amount of the formulation can be spat out by the animals. However, the application of the formulation still can be hindered if the animals are keeping free range (not in the boxes) during the treatment due to the preiously mentioned reasons.

The other way to solve the problem of the unstable nature of EOs, especially in ruminants, represent the use of encapsulation techniques that can protect the main active ingredients that are sensitive to various factors (oxygen, light and moisture), and prevent interaction with other compounds. In this way, the stability and bioavailability of EOs can be further increased, while reducing toxicity, volatility, odour and taste. Also important, encapsulation enables a controlled release of the active ingredients of EOs along the gastrointestinal tract [51, 58, 59]. As the dose tested did not cause any adverse effects in the animals, increasing the dose or multiple applications during several consecutive days could also be an option to improve efficacy under field conditions. Alternatively, other forms of EO application such as drenching [60] or the use of lick blocks with herbal compounds [61] should also be considered. Ultimately, EOs, which have a certain but not required efficacy of ≥ 90% [62], can still be a valuable component of an integrated approach to sustainable control of GINs along with other methods [63].

According to the results of GC–MS analysis (Table 1, Figure 1), the most important compounds responsible for the anthelmintic properties of winter savory are p-cymene, carvacrol and γ-terpinene. In a study by André et al. [64], the isolated carvacrol showed high anthelmintic potential in various in vitro tests, including the larval development test (LDT) and the adult worm motility test (AMT) in addition to the EHT. This indicates that this compound is effective against different parasite stages. Moreover, in the same study, its acetylated derivative reduced the group EPG by 65.9% at D16 after treatment in the FECRT. Its strong anthelmintic activity was also confirmed in a study by Katiki et al. [65], in which it showed one of the highest ovicidal effects against *H. contortus* eggs compared to other compounds. p-Cymene and γ-terpinene were not individually tested for their anthelmintic activity against GINs, but were present in some EOs with this property: p-cymene at 22.56% in *Alpinia zerumbet* [66] and at 23.76% in *Thymus vulgaris* [67] as well as γ-terpinene at 20.15% in *Melaleuca alternifolia* [68] and at 11.42% in *Citrus aurantifolia* [43].

Not only the presence, but also the percentage of the compounds in the EOs is important for the pharmacological effects. Thus, these three compounds were also the main constituents of the previously tested oregano EO [35], but in a different ratio: carvacrol 76.21%, p-cymene 12.57% and γ-terpinene 2.63%. However, oregano showed a superior effect with an total group EPG reduction of 43.21% and 60.13% at D7 and D14 after treatment, respectively. This could indicate a superior activity of carvacrol compared to p-cymene, although this statement needs to be confirmed by individual testing and comparison of these compounds. Indeed, individual compounds can differ considerably in their anthelmintic activity [65]. Moreover, the dominance of one compound and its appropriate abundance (at least > 50%) with the simultaneous presence of other compounds but in lower percentages, may lead to high activity, as shown by the results of a previously conducted study with eleven EO tested in vitro [48]. These considerations could be useful for the future development of herbal anthelmintics.

Preliminary toxicity studies (Tables 4 and 5) indicate that the use of winter savory is safe in practice, at least as far as short-term side effects are concerned (14 days after treatment). Namely, no changes in feeding, defecation or animal behaviour were observed after the treatments. None of the tested haematological parameters were significantly altered (*p* > 0.05) and remained within the reference range, except for the parameters that were also outside this range before treatment. In fact, the administered *S. montana* EO, as well as albendazole, reduced to some extent the signs of anaemia observed in the animals by slightly increasing the levels of RBC, Hgb and HCT. However, this can be attributed to the effect of the tested EO on the parasite load and the resulting improvement in the clinical aspects of the animals, including the degree of anaemia. For the biochemical parameters reflecting the effect of the administered EO on renal (urea and creatinine) and hepatic (AST and GGT) function, the absence of any statistical changes (*p* > 0.05) and, in the case of GGT, even significantly decreased values (*p* < 0.05), indicates that the administered EO did not affect their function. The same trend was also observed in our previous studies with the EOs of oregano [35] and peppermint [36], in which the same preliminary toxicity studies were conducted, with no toxic effects observed in any of the animals tested at the administered dose (150 mg/kg bw, intraruminal). In other similar studies such as those conducted by Katiki et al. [69, 70], where the EO of lemongrass (*Cymbopogon schoenanthus*) was administered orally at doses of 180 and 360 mg/kg, and an encapsulated combination of anethole and carvone that was administered through the feed at doses of 20 and 50 mg/kg, the results showed that these plant products are safe for the lambs. However, although many of the EOs are currently considered safe for use in animals intended for human consumption [71], the exact studies confirming this fact are still scarce [25], suggesting that toxicity studies should be a regular part of studies aimed at evaluating the possibility of using plant products in animals.

As shown in Tables 6 and 7, the results of the coproculture study revealed the presence of four GINs genera in both farms, namely *Haemonchus*, *Trichostrongylus*, *Teladorsagia* and *Chabertia*, which are regularly found in this region [6, 48]. Apart from the differences in terms of genera representation between the two farms tested (*p* < 0.05), which is to be expected as these are two different farms and it is not important for the results obtained, no statistically significant difference in representation was observed for any of the genera before and after treatment in either farm (*p* > 0.05), which indicates that *S. montana* EO is not specific to a single genus. However, some other in vivo studies [42, 72] have shown that natural products, administered perorally, can have different effects on individual GINs observed on FECRT or CET, usually the highest one against *H. contortus* in comparison with e.g., *Trichostrongylus* spp. and *O. columbianum*. These results can be explained by the location of the gastrointestinal tract where various GINs are found, with the applied EOs acting mainly on *H. contortus* and other nematodes parasitizing in the abomasum, where the bioavailability of the EO active ingredients is higher after peroral administration compared to the small and large intestine. Further studies evaluating the efficacy against different GINs separately should also be conducted with *S. montana* EO.

Plant-based formulations have many advantages that can be exploited for their use in animals for various indications, including the control of GINs [73–75]. The compounds that compose them have a wide variety of pharmacological effects, belong to different chemical groups with possibly different mechanisms of action, and have therefore already shown the effect against various parasite stages of GINs [24, 25]. This may also contribute to their less susceptibility to resistance development in comparison with synthetic agents [43, 76]. Botanical drugs are also considered safer than synthetic, whereby environmental aspect and public health also favouring natural drugs due to their biodegradability, to challenge the residue problem [13, 16, 77]. Finally, the price of plant drugs is also considered reasonable and even cheaper than that of commercial drugs, so the procurement of these medicines should not be a problem, especially in countries with developed biodiversity [43], as in the case of *S. montana* in Serbia [78]. For these reasons, the possibility of using plant-based drugs against parasites is the subject of a large and increasing number of studies in recent times, attracting a growing number of researchers worldwide [25, 79–83], with promising results being obtained in most of these studies.

Novel anthelmintics can be used in different ways to control sheep GINs. They can be used independently, but only if they achieve sufficient efficacy under field conditions. Although a threshold of 95% is used to assess efficacy and the development of potential resistance to commercial anthelmintics in ruminants [12], some authors recommend a minimum efficacy of 90% in FECRT for new anthelmintic agents [62, 84]. Thus, *S. montana* as well as EOs from other studies, are not yet suitable for independent use in the doses, formulations and route of administration examined. However, other alternative methods such as genetic selection of animals that are naturally resistant to nematodes [85], pasture management and nutritional manipulations [86], biological control methods (direct – use of nematophagous fungi, bacteria or even other nematodes or indirect – use of deep buntle or earthworms) [87] and development of vaccines [88] also have some limitations despite their numerous advantages. Finally, the exclusive use of commercial drugs is also no longer suitable due to the development and spread of resistance, as well as residue issues that concerns public health and environment [11, 13, 89], as already mentioned. Therefore, there is a broad consensus that integrated parasite management, which refers to the use of several appropriate methods of parasite control, represent the most appropriate solution for the future management of GINs in sheep [90–93]. It has been shown that carvacrol, one of the main constituents of *S. montana* EO, can enhance the effect of drugs that are agonists of the nAChR (imidazothiazoles) or the agonists of GABA receptors (avermectins and piperazine) [94, 95]. Studies with other plant species showed that the combination of e.g., ethanol extract of *Ananas comosu*s with the fungal product *Clonostachys rosea* effectively reduced faecal egg and larva counts, larval development and the number of infective larvae (L3) in the pasture [60]. These examples have shown that various plant-based anthelmintics, including EOs and their active ingredients, may represent a valuable source in combination with commercial drugs, or other mentioned alternatives.

## Conclusion

The results of the efficacy and preliminary toxicity tests conducted in this study indicate that the EO of *S. montana* has the potential to be used against GINs in sheep. The in vivo efficacy should be further improved by increasing the dose, different way of use including multiple application or the use of encapsulation technique. Nevertheless, the use of this oil, in association with other methods, may help to reduce the current frequency of treatments with chemotherapeutic agents to slow down the development of AR, and reduce the negative effects of these drugs. Further studies of *S. montana* EO with performing other testing, including those against individual GIN species (especially those that are resistant to existing drugs), should be performed.

## Data Availability

The data contained in this manuscript were part of the PhD thesis of the first and corresponding author, Dr. Filip Štrbac. Printed version of the dissertation is available in the library of Faculty of Agriculture, University of Novi Sad, Serbia, where the thesis was defended. In electronic form, it is available on the repository of the institution affiliated with the first author - Institute for Multidisciplinary Research, University of Belgrade, Serbia at the following link: https://rimsi.imsi.bg.ac.rs/handle/123456789/2459. Also, previous version of this manuscript is available online as a preprint on the following link: https://www.researchsquare.com/article/rs-4576907/v1.

## References

[1] Desalegn C, Berhanu G. Assessment of the epidemiology of the gastrointestinal tract nematode parasites in sheep in Toke Kutaye, West Shoa Zone. Ethiopia Vet Med (Auckl). 2023;14:177–83. 10.2147/VMRR.S427828. 37808535 10.2147/VMRR.S427828 PMC10559793

[2] van Dijk M, Morley T, Rau ML, Saghai Y. A meta-analysis of projected global food demand and population at risk of hunger for the period 2010–2050. Nat Food. 2021;2:494–501. 10.1038/s43016-021-00322-9. 37117684 10.1038/s43016-021-00322-9

[3] Tachack EB, Oviedo-Socarrás T, Pastrana MO, Pérez-Cogollo LC, Bernavides YH, Pinto CR, et al. Status of gastrointestinal nematode infections and associated epidemiological factors in sheep from Córdoba. Colombia Trop Anim Health Prod. 2022;54:171. 10.1007/s11250-022-03170-2. 35471467 10.1007/s11250-022-03170-2 PMC9042984

[4] Maurizio A, Perrucci S, Tamponi C, Scala A, Cassini R, Rinaldi L, et al. Control of gastrointestinal helminths in small ruminants to prevent anthelmintic resistance: the Italian experience. Parasitology. 2023;150:1105–18. 10.1017/S0031182023000343. 37039466 10.1017/S0031182023000343 PMC10801368

[5] Abubakar M, Oneeb M, Rashid M, Ashraf K, Chisti GA, Awan F, et al. *In vitro* anthelmintic efficacy of three plant extracts against various developmental stages of *Haemonchus contortus*. Pak Vet J. 2024;44:238–43. 10.29261/pakvetj/2024.174

[6] Bosco A, Kießler J, Amadesi A, Varady M, Hinney B, Ianniello D, et al. The threat of reduced efficacy of anthelmintics against gastrointestinal nematodes in sheep from an area considered anthelmintic resistance-free. Parasit Vectors. 2020;13:457. 10.1186/s13071-020-04329-2. 32907633 10.1186/s13071-020-04329-2 PMC7487796

[7] Charlier J, Rinaldi L, Musella V, Ploeger HW, Chartier C, Rose Vineer H, et al. Initial assessment of the economic burden of major parasitic helminth infections to the ruminant livestock industry in Europe. Prev Vet Med. 2020;182: 105103. 10.1016/j.prevetmed.2020.105103. 32750638 10.1016/j.prevetmed.2020.105103

[8] Fox NJ, Smith LA, Houdijk JGM, Athanasiadou S, Hutchings MR. Ubiquitous parasites drive a 33% increase in methane yield from livestock. Int J Parasitol. 2018;48:1017–21. 10.1016/j.ijpara.2018.06.001. 30107148 10.1016/j.ijpara.2018.06.001

[9] Rose Vineer H, Morgan ER, Hertzberg H, Bartley DJ, Bosco A, Charlier J, et al. Increasing importance of anthelmintic resistance in European livestock: creation and meta-analysis of an open database. Parasite. 2020;27:69. 10.1051/parasite/2020062. 33277891 10.1051/parasite/2020062 PMC7718593

[10] Ahbara AM, Rouatbi M, Gharbi M, Rekik M, Haile A, Rischkowsky B, et al. Genome-wide insights on gastrointestinal nematode resistance in autochthonous Tunisian sheep. Sci Rep. 2021;11:9250. 10.1038/s41598-021-88501-3. 33927253 10.1038/s41598-021-88501-3 PMC8085236

[11] de Agüero VCG, Valderas-García E, del Palacio LG, Giráldez FJ, Balaña-Fouce R, Martínez-Valladares M. Secretory IgA as biomarker for gastrointestinal nematodes natural infection in different breed sheep. Animals. 2023;13:2189. 10.3390/ani13132189. 37443987 10.3390/ani13132189 PMC10339930

[12] Kaplan RM. Biology, epidemiology, diagnosis and management of anthelmintic resistance in gastrointestinal nematodes of livestock. Vet Clin North Am Food Anim Pract. 2020;36:17–30. 10.1016/j.cvfa.2019.12.001. 32029182 10.1016/j.cvfa.2019.12.001

[13] Castagna F, Bava R, Musolino V, Piras C, Cardamone A, Carresi C, et al. Potential new therapeutic approaches based on Punica granatum fruits compared to synthetic anthelmintics for the sustainable control of gastrointestinal nematodes in sheep. Animals. 2022a;12:2883. 10.3390/ani12202883. 10.3390/ani12202883 PMC9598348 36290268

[14] Ragusa M, Miceli N, Piras C, Bosco A, Castagna F, Rinaldi L, et al. *In vitro* anthelmintic activity of Isatis tinctoria extracts against ewes’ gastrointestinal nematodes (GINs), a possible application for animal welfare. Vet Sci. 2022;9:129. 10.3390/vetsci9030129. 35324857 10.3390/vetsci9030129 PMC8949818

[15] Varshney B, Malik S, Singh A, Mehta N. Role of medicinal plants and herbs in veterinary medicine. In: Singh A, editor. Handbook of Advanced phytochemicals and plant-based drug discovery. IGI Global: 2022., Chapter 3, p. 32–48. 10.4018/978-1-6684-5129-8.

[16] Romero B, Susperregui J, Sahagún AM, Diez MJ, Fernández N, García JJ, et al. Use of medicinal plants by veterinary practitioners in Spain: A cross-sectional survey. Front Vet Sci. 2022;9:1060738. 10.3389/fvets.2022.1060738. 36590819 10.3389/fvets.2022.1060738 PMC9797804

[17] Shao Y, Wang Y, Yuan Y, Xie Y. A systematic review on antibiotics misuse in livestock and aquaculture and regulation implications in China. Sci Total Environ. 2021;798: 149205. 10.1016/j.scitotenv.2021.149205. 34375247 10.1016/j.scitotenv.2021.149205

[18] Bava R, Castagna F, Ruga S, Nucera S, Caminiti R, Serra M, et al. Plants and their derivatives as promising therapeutics for sustainable control of honeybee (*Apis mellifera*) Pathogens. 2023;12:1260. 10.3390/pathogens12101260 10.3390/pathogens12101260 PMC10610010 37887776

[19] Napoli E, Di Vito M. Toward a new future for essential oils. Antibiotics. 2021;10:207. 10.3390/antibiotics10020207. 33669818 10.3390/antibiotics10020207 PMC7923015

[20] Issa (2024) Evaluation the antimicrobial activity of essential oils against veterinary pathogens, multidrug-resistant bacteria and Dermatophytes. Pak Vet J. 2024;4:260–5. 10.29261/pakvetj/2024.165

[21] Ebani VV, Mancianti F. Use of essential oils in veterinary medicine to combat bacterial and fungal infections. Vet Sci. 2020;7:193. 10.3390/vetsci7040193. 33266079 10.3390/vetsci7040193 PMC7712454

[22] Mucha W, Witkowska D. The applicability of essential oils in different stages of production of animal-based foods. Molecules. 2021;26:3798.10.3390/molecules26133798 34206449 10.3390/molecules26133798 PMC8270267

[23] Ratajac R, Pavličević A, Petrović J, Stojanov I, Orčić D, Štrbac F, et al. *In vitro* Evaluation of acaricidal efficacy of selected essential oils against *Dermanyssus gallinae*. Pak Vet J. 2024;44:93–8. 10.29261/pakvetj/2023.123

[24] André WPP, Ribeiro WLC, de Oliveira LMB, Macedo ITF, Rondon FCR, Bevilaqua CML. Essential oils and their bioactive compounds in the control of gastrointestinal nematodes of small ruminants. Acta Sci Vet. 2018;46:1522. 10.22456/1679-9216.81804

[25] Štrbac F, Bosco A, Pušić I, Stojanović D, Simin N, Cringoli G, et al. The use of essential oils against sheep gastrointestinal nematodes. In: RZ Abbas, A Khan, P Liu, and MK Saleemi, editors. Animal Health Perspectives, Vol 1. Unique Scientific Publishers, Faisalabad, Pakistan; 2022a. p. 86–94. 10.47278/book.ahp/2022.12.

[26] Aćimović M, Šovljanski O, Pezo L, Travičić V, Tomić A, Zheljazkov VD, et al. Variability in biological activities of *Satureja montana* Subsp. *montana* and Subsp. *variegata* based on different extraction methods. Antibiotics. 2022a;11:1235. 10.3390/antibiotics11091235. 10.3390/antibiotics11091235 PMC9495055 36140014

[27] Aćimović M, Todosijević M, Varga A, Kiprovski B, Tešević V, Čabarkapa I, et al. Bioactivity of essential oils from cultivated winter savory, sage and hyssop. Lek Sirov. 2019;39:11–7. 10.5937/leksir1939011A.

[28] Tomaselli V, Silletti G, Forte L. A new association of *Satureja montana* L. subsp. *montana* dominated garrigues in Puglia (SE Italy). Plant Sociol. 2021;58:1–14. 10.3897/pls2021582/01

[29] Maccelli A, Vitanza L, Imbriano A, Fraschetti C, Filippi A, Goldoni P, et al. *Satureja montana* L. essential oils: Chemical profiles/phytochemical screening, antimicrobial activity and O/W nanoemulsion formulations. Pharmaceutics. 2020;12:7. 10.3390/pharmaceutics12010007 10.3390/pharmaceutics12010007 PMC7022231 31861717

[30] Matejić J, Stefanović N, Ivković M, Živanović N, Marin P, Džamić A. Traditional uses of autochthonous medicinal and ritual plants and other remedies for health in Eastern and South-Eastern Serbia. J Ethnopharmacol. 2020;261: 113186. 10.1016/j.jep.2020.113186. 32730888 10.1016/j.jep.2020.113186

[31] Aćimović M, Šovljanski O, Šeregelj V, Pezo L, Zheljazkov VD, Ljujić J, et al. Chemical composition, antioxidant, and antimicrobial activity of *Dracocephalum moldavica* L. essential oil and hydrolate. Plants. (Basel) 2022b;11:941. 10.3390/plants11070941 10.3390/plants11070941 PMC9002726 35406925

[32] Knežević P, Aleksić V, Simin N, Svirčev E, Petrović A. Mimica-Dukić N (2016) Antimicrobial activity of *Eucalyptus camaldulensis* essential oils and their interactions with conventional antimicrobial agents against multi-drug resistant *Acinetobacter baumannii*. J Ethnopharmacol. 2016;178:125–36. 10.1016/j.jep.2015.12.008. 26671210 10.1016/j.jep.2015.12.008

[33] Adams RP. Identification of essential pil components by gas chromatography/mass spectrometry, 4th ed. Allured Business Media: Carol Stream, IL, USA; 2012.

[34] Bosco A, Maurelli MP, Ianniello D, Morgoglione ME, Amadesi A, Coles GC, et al. The recovery of added nematode eggs from horse and sheep faeces by three methods. BMC Vet Res. 2018;14:7. 10.1186/s12917-017-1326-7. 29304858 10.1186/s12917-017-1326-7 PMC5756441

[35] Štrbac F, Krnjajić S, Maurelli MP, Stojanović D, Simin N, Orčić D, et al. A potential anthelmintic phytopharmacological source of *Origanum vulgare* (L.) essential oil against gastrointestinal nematodes of sheep. Animals. 2023a;13:45. 10.3390/ani13010045 10.3390/ani13010045 PMC9817997 36611652

[36] Štrbac F, Krnjajić S, Stojanović D, Ratajac R, Simin N, Orčić D, et al. *In vitro* and *in vivo* anthelmintic efficacy of peppermint (*Mentha x piperita* L.) essential oil against gastrointestinal nematodes of sheep. Front Vet Sci. 2023b;10:1232570. 10.3389/fvets.2023.1232570 10.3389/fvets.2023.1232570 PMC10472939 37662995

[37] Cringoli G, Maurelli MP, Levecke B, Bosco A, Vercruysse J, Utzinger J, et al. The Mini-FLOTAC technique for the diagnosis of helminth and protozoan infections in humans and animals. Nat Protoc. 2017;12:1723–32. 10.1038/nprot.2009.235. 28771238 10.1038/nprot.2017.067

[38] Sajid M, Naqvi SAH, Riaz M, Umar UUD, Nasreen N, Khan A, et al. Molecular detection of Babesia ovis and blood parameters’ investigation reveal hematological and biochemical alterations in babesiosis-infected Lohi sheep in Multan. Pakistan Open Vet J. 2023;13:1400–8. 10.5455/OVJ.2023.v13.i11.2. 38107231 10.5455/OVJ.2023.v13.i11.2 PMC10725295

[39] Ministry of Agriculture, Fisheries and Food (MAFF). Grande-Bretagne, Manual of Veterinary Parasitological Laboratory Techniques. London: ADAS, HM Stationery Office; 1986.

[40] van Wyk JA, Mayhew E. Morphological identification of parasitic nematode infective larvae of small ruminants and cattle: A practical lab guide. Onderstepoort J Vet Res. 2013;80:539. https://hdl.handle.net/10520/EJC134539 10.4102/ojvr.v80i1.539 23718204

[41] Coles GC, Bauer C, Borgsteede FH, Geerts S, Klei TR, Taylor MA, et al. World association for the advencement of veterinary parasitology (W.A.A.V.P.) methods for the detection of anthelmintic resistance in nematodes of veterinary importance. Vet Parasitol. 1992;44:35–44. 10.1016/0304-4017(92)90141-U 10.1016/0304-4017(92)90141-u 1441190

[42] Pinto NB, de Castro LM, Azambuja RHM, Capella GDA, de Moura MQ, Terto WD, et al. Ovicidal and larvicidal potential of *Rosmarinus officinalis* to control gastrointestinal nematodes of sheep. Rev Bras Parasitol Vet. 2019;28:807–11. 10.1590/S1984-29612019060. 31483032 10.1590/S1984-29612019060

[43] Ferreira LE, Benincasa BI, Fachin AL, Contini SHT, França SC, Chagas ACS, et al. Essential oils of *Citrus aurantifola*, *Anthemis nobile* and *Lavandula officinalis*: *In vitro* anthelmintic activities against *Haemonchus contortus*. Parasit Vectors. 2018;11:269. 10.1186/s13071-018-2849-x 29695271 10.1186/s13071-018-2849-x PMC5918559

[44] Macedo ITF, de Oliveira LMB, Andre WBP, Filho JVA, dos Santos JML, Rondon FCM, et al. Anthelmintic effect of *Cymbopogon citratus* essential oil and its nanoemulsion on sheep gastrointestinal nematodes. Rev Bras Parasitol Vet. 2019;28:522–7. 10.1590/S1984-29612019065. 31483036 10.1590/S1984-29612019065

[45] Várady M, Čudeková P, Čorba J. *In vitro* detection of benzimidazole resistance in *Haemonchus contortus*: Egg hatch test versus larval development test. Vet Parasitol. 2007;149:104–10. 10.1016/j.vetpar.2007.07.011. 17697753 10.1016/j.vetpar.2007.07.011

[46] Bhinsara DB, Sankar M, Desai DN, Hasnani JJ, Patel PV, Hirani ND, et al. Anthelmintic drug-resistant detection methods: A brief overview. Int J Adv Biol Biomed Res. 2018;8:433–7.

[47] Babják M, Königová A, Dolinská MU, Kupčinskas T, Vadlejch J, von Samson-Himmelstjerna G, et al. Does the *in vitro* egg hatch test predict the failure of benzimidazole treatment in *Haemonchus contortus*? Parasite. 2021;28:62. 10.1051/parasite/2021059. 34410223 10.1051/parasite/2021059 PMC8375488

[48] Štrbac F, Bosco A, Maurelli MP, Ratajac R, Stojanović D, Simin N, et al. Anthelmintic properties of essential oils to control gastrointestinal nematodes in sheep - *In vitro* and *in vivo* studies. Vet Sci. 2022b;9:93. 10.3390/vetsci9020093. 35202346 10.3390/vetsci9020093 PMC8880401

[49] Berrouet C, Dorilas N, Rejniak KA, Tuncer N. Comparison of drug inhibitory effects (IC_50_) in monolayer and spheroid cultures. Bull Math Biol. 2020;82:68. 10.1007/s11538-020-00746-7. 32495209 10.1007/s11538-020-00746-7 PMC9773863

[50] Fokou JBH, Dongmo PMJ, Boyom FF. Essential oil’s chemical composition and pharmacological properties. In: El-Shemy H, editor. Essential oils - oils of nature. IntechOpen: London, UK; 2020. Chapter 2. 10.5772/intechopen.86573

[51] Cimino C, Maurel OM, Musumeci T, Bonaccorso A, Drago F, Souto EMB, et al. Essential oils: Pharmaceutical applications and encapsulation strategies into lipid-based delivery systems. Pharmaceutics. 2021;13:327. 10.3390/pharmaceutics13030327. 33802570 10.3390/pharmaceutics13030327 PMC8001530

[52] Hendawy SHM. Immunity to gastrointestinal nematodes in ruminants: effector cell mechanisms and cytokines. J Parasit Dis. 2018;42:471–82. 10.1007/s12639-018-1023-x. 30538343 10.1007/s12639-018-1023-x PMC6261135

[53] Kaplan RM, Denwood MJ, Nielsen MK, Thamsborg SM, Torgersen PR, Gilleard JS, et al. World Association for the Advancement of Veterinary Parasitology (W.A.A.V.P.) guideline for diagnosing anthelmintic resistance using the faecal egg count reduction test in ruminants, horses and swine. Vet Parasitol. 2023;318:109936. 10.1016/j.vetpar.2023.109936 10.1016/j.vetpar.2023.109936 37121092

[54] Love JW, Kelly LA, Lester HE, Nanjiani I, Taylor MA, Robertson C. Investigating anthelmintic efficacy against gastrointestinal nematodes in cattle by considering appropriate probability distributions for faecal egg count data. Int J Parasitol Drugs Drug Resist. 2017;7:71–82. 10.1016/j.ijpddr.2017.01.002. 28161555 10.1016/j.ijpddr.2017.01.002 PMC5293727

[55] Denwood MJ, Kaplan RM, McKendrick IJ, Thamsborg SM, Nielsen MK, Levecke B. A statistical framework for calculating prospective sample sizes and classifying efficacy results for faecal egg count reduction tests in ruminants, horses and swine. Vet Parasitol. 2023;314: 109867. 10.1016/j.vetpar.2022.109867. 36621042 10.1016/j.vetpar.2022.109867

[56] Nehme R, Andrés S, Pereira RB, Jemaa MB, Bouhallab S, Ceciliani F, et al. Essential oils in livestock: From health to food quality. Antioxidants. 2021;10:330. 10.3390/antiox10020330. 33672283 10.3390/antiox10020330 PMC7926721

[57] Hoste H, Torres-Acosta JF, Alonso-Diaz MA, Brunet S, Sandoval-Castro C, Adote SH. Identification and validation of bioactive plants for the control of gastrointestinal nematodes in small ruminants. Trop Biomed. 2008;25:56–72. 18414378

[58] Sousa VI, Parente JF, Marques JF, Forte MA, Tavares CJ. Microencapsulation of essential oils: A review. Polymer. 2022;14:1730. 10.3390/polym14091730. 10.3390/polym14091730 PMC9099681 35566899

[59] Yammine J, Chihib NE, Gharsallaoui A, Ismail A, Karam L. Advances in essential oils encapsulation: development, characterization and release mechanisms. Polym Bull. 2024;81:3837–82. 10.1007/s00289-023-04916-0.

[60] Ahmed M, Laing MD, Nsahlai IV. A new control strategy for nematodes of sheep using chlamydospores of a fungus, *Clonostachys rosea f. rosea*, and an ethanolic extract of a plant, *Ananas comosus*. Biocontrol Sci Technol. 2014;24:860–71. 10.1080/09583157.2014.897304

[61] Junkuszew A, Milerski M, Bojar W, Szczepaniak K, Le Scouarnec J, Tomczuk K, et al. Effect of various antiparasitic treatments on lamb growth and mortality. Small Rumin Res. 2015;123:306–13. 10.1016/j.smallrumres.2014.11.019.

[62] Burden DJ, Bartley DJ, Besier RB, Claerebout E, Elliott TP, Höglund J, et al. World Association for the Advancement of Veterinary Parasitology (W.A.A.V.P.): Third edition of the guideline for evaluating efficacy of anthelmintics in ruminants (bovine, ovine, caprine). Vet Parasitol. 2024;329:110187. 10.1016/j.vetpar.2024.110187 10.1016/j.vetpar.2024.110187 38728835

[63] Macedo ITF, Bevilaqua CML, de Oliveira LMB, Camurça-Vasconcelos ALF, Vieira LDS, Oliveira FR, et al. Anthelmintic effect of *Eucalyptus staigeriana* essential oil against goat gastrointestinal nematodes. Vet Parasitol. 2010;173:93–8. 10.1016/j.vetpar.2010.06.004. 20609526 10.1016/j.vetpar.2010.06.004

[64] André WPP, Ribeiro WLC, Cavalcante GS, dos Santos JML, Macedo ITF, de Paula HCB, et al. Comparative efficacy and toxic effects of carvacryl acetate and carvacrol on sheep gastrointestinal nematodes and mice. Vet Parasitol. 2016;218:52–8. 10.1016/j.vetpar.2016.01.001. 26872928 10.1016/j.vetpar.2016.01.001

[65] Katiki LM, Barbieri AME, Araujo RC, Veríssimo CJ, Louvandini H, Ferreira JFS. Synergistic interaction of ten essential oils against *Haemonchus contortus in vitro*. Vet Parasitol. 2017;243:47–51. 10.1016/j.vetpar.2017.06.008. 28807309 10.1016/j.vetpar.2017.06.008

[66] Macedo ITF, de Oliveira LMB, Camurça-Vasconcelos ALF, Ribeiro WLC, dos Santos JML, de Morais SM, et al. *In vitro* effects of *Coriandrum sativum*, *Tagetes minuta*, *Alpinia zerumbet* and *Lantana camara* essential oils on *Haemonchus contortus*. Rev Bras Parasitol Vet. 2013;22:463–9. 10.1590/S1984-29612013000400004. 24473869 10.1590/S1984-29612013000400004

[67] Ferreira LE, Benincasa BI, Fachin AL, França SC, Contini SSHT, Chagas ACS, et al. *Thymus vulgaris* L. essential oil and its main component thymol: Anthelmintic effects against *Haemonchus contortus* from sheep. Vet Parasitol. 2016;228:70–6. 10.1016/j.vetpar.2016.08.011 10.1016/j.vetpar.2016.08.011 27692335

[68] Grando TH, de Sá MF, Baldissera MD, Oliveira CB, de Souza ME, Raffin RP, et al. *In vitro* activity of essential oils of free and nanostructured *Melaleuca alternifolia* and of terpinen-4-ol on eggs and larvae of *Haemonchus contortus*. J Helminthol. 2015;90:377–82. 10.1017/S0022149X15000401. 26096177 10.1017/S0022149X15000401

[69] Katiki LM, Chagas ACS, Takahira RK, Juliani HR, Ferreira JFS, Amarante AFT. Evaluation of *Cymbopogon schoenanthus* essential oil in lambs experimentally infected with *Haemonchus contortus*. Vet Parasitol. 2012;186:312–8. 10.1016/j.vetpar.2011.12.003. 22206645 10.1016/j.vetpar.2011.12.003

[70] Katiki LM, Araujo RC, Ziegelmeyer L, Gomes ACP, Gutmanis G, Rodrigues L, et al. Evaluation of encapsulated anethole and carvone in lambs artificially- and naturally-infected with *Haemonchus contortus*. Exp Parasitol. 2019;197:36–52. 10.1016/j.exppara.2019.01.002. 30633915 10.1016/j.exppara.2019.01.002

[71] Wells CW. Effects of essential oils on economically important characteristics of ruminant species: A comprehensive review. Anim Nutr. 2024;16:1–10. 10.1016/j.aninu.2023.05.017. 38131027 10.1016/j.aninu.2023.05.017 PMC10731003

[72] Camurça-Vasconcelos ALF, Bevilaqua CML, Morais SM, Maciel MV, Costa CTC, Macedo ITF, et al. Anthelmintic activity of *Lippia sidoides* essential oil on sheep gastrointestinal nematodes. Vet Parasitol. 2008;154:167–70. 10.1016/j.vetpar.2008.02.023. 18423877 10.1016/j.vetpar.2008.02.023

[73] Bava R, Castagna F, Piras C, Palma E, Cringoli G, Musolino V, et al. *In vitro* evaluation of acute toxicity of five *Citrus* spp. essential oils towards the parasitic mite *Varroa destructor*. Pathogens. 2021;10:1182. 10.3390/pathogens10091182 10.3390/pathogens10091182 PMC8466118 34578214

[74] Castagna F, Piras C, Palma E, Musolino V, Lupia C, Bosco A, et al. Green veterinary pharmacology applied to parasite control: Evaluation of *Punica granatum*, *Artemisia campestris*, *Salix caprea* aqueous macerates against gastrointestinal nematodes of sheep. Vet Sci. 2021;8:237. 10.3390/vetsci8100237. 34679067 10.3390/vetsci8100237 PMC8539373

[75] Castagna F, Bava R, Piras C, Carresi C, Musolino V, Lupia C, et al. Green veterinary pharmacology for honey bee welfare and health: *Origanum heracleoticum* L. (Lamiaceae) essential oil for the control of the *Apis mellifera* varroatosis. Vet Sci. 2022b;9:124. 10.3390/vetsci9030124 10.3390/vetsci9030124 PMC8953610 35324852

[76] Borges DGL, Borges FDA. Plants and their medicinal potential for controling gastrointestinal nematodes in ruminants. Nematoda. 2016;3: e92016. 10.4322/nematoda.00916

[77] Veerakumari L. Botanical anthelmintics. Asian J. Sci Technol. 2015;6:1881–94.

[78] Šubarević N, Stevanović O, Petrujkić B. Use of phytotherapy as a form of ethnoveterinary medicine in the area of Stara planina mountain in Serbia. Acta Med Hist Adriat. 2015;13:75–94. 26203540

[79] Boyko O, Brygadyrenko V. Survival of nematode larvae after treatment with eugenol, isoeugenol, thymol, and carvacrol. Front Biosci. 2023;15:25. 10.31083/j.fbe1504025 10.31083/j.fbe1504025 38163936

[80] Dağ ŞRO, Erez MS, Kozan E, Özkan AMG, Çankaya IIT. *In vitro* anthelmintic activity of five different *Artemisia* L. species growing in Türkiye. Pak Vet J. 2023;43:771–7. 10.29261/pakvetj/2023.087

[81] Osório TM, Menezes LDM, Martins AA, Schimidt D, Pretto MM, da Rosa KB, et al. Essential oils against gastrointestinal nematodes in sheep *in vitro* and chemical composition of those plants. Ciênc Nat. 2023;45: e25. 10.5902/2179460X71665.

[82] Belga FN, Waindok P, Raulf MK, Jato J, Orman E, Rehbein S, et al. Phytochemical analysis and anthelmintic activity of *Combretum mucronatum* leaf extract against infective larvae of soil-transmitted helminths including ruminant gastrointestinal nematodes. Parasit Vectors. 2024;17:99. 10.1186/s13071-024-06194-9. 38429804 10.1186/s13071-024-06194-9 PMC10905826

[83] Santiago-Figueroa I, González-Cortazar M, Estrada-Flores JG, Cuéllar-Ordaz JA, López-Arellano ME, González-Reyes FJ, et al. Synergistic interaction effect of *Artemisia cina* n-hexane extract and *Tagetes lucida* ethyl acetate extract on *Haemonchus contortus*. Acta Parasitol. 2024;69:1132–40. 10.1007/s11686-024-00839-6. 38568361 10.1007/s11686-024-00839-6 PMC11182837

[84] Geurden T, Smith ER, Vercruysse J, Yazwinski T, Settje T, Nielsen MK. World association for the advancement of veterinary parasitology (WAAVP) guideline for the evaluation of the efficacy of anthelmintics in food-producing and companion animals: general guidelines. Vet Parasitol. 2022;304: 109698. 10.1016/j.vetpar.2022.109698. 35305843 10.1016/j.vetpar.2022.109698

[85] Tsukahara Y, Gipson TA, Hart SP, Dawson L, Wang Z, Puchala R, et al. Genetic selection for resistance to gastrointestinal parasitism in meat goats and hair sheep through a performance test with artificial infection of *Haemonchus contortus*. Animals. 2021;11:1902. 10.3390/ani11071902. 34206774 10.3390/ani11071902 PMC8300302

[86] Yoshihara Y, Saiga C, Tamura T, Kinugasa T. Relationships between sheep nematode infection, nutrition, and grazing behavior on improved and semi-natural pastures. Vet Anim Sci. 2023;19: 100278. 10.1016/j.vas.2022.100278. 36561431 10.1016/j.vas.2022.100278 PMC9764242

[87] Szewc M, De Waal T, Zintl A. Biological methods for the control of gastrointestinal nematodes. Vet J. 2021;268: 105602. 10.1016/j.tvjl.2020.105602. 33468301 10.1016/j.tvjl.2020.105602

[88] Liu H, Zhang Y, Liu F, Ye L, Liu X, Wang C, et al. Progress and challenges for developing vaccines against gastrointestinal nematodes of ruminants. Vet Vacc. 2023;100041. 10.1016/j.vetvac.2023.100041

[89] Maestrini M, Forzato C, Mancini S, Pieracci Y, Perrucci S. *In vitro* anthelmintic activity of sea buckthorn (*Hippophae rhamnoides*) berry juice against gastrointestinal nematodes of small ruminants. Biology. 2022;11:825. 10.3390/biology11060825. 35741346 10.3390/biology11060825 PMC9219796

[90] Maqbool I, Wani ZA, Shahardar RA, Allaie IM, Shah MM. Integrated parasite management with special reference to gastro-intestinal nematodes. J Parasit Dis. 2017;41:1–8. 10.1007/s12639-016-0765-6. 28316380 10.1007/s12639-016-0765-6 PMC5339188

[91] Tariq KA. Anthelmintics and emergence of anthelmintic resistant nematodes in sheep: need of an integrated nematode management. Int J Vet Sci Anim Husb. 2017;2:13–9.

[92] Mondragón-Ancelmo J, Olmedo-Juárez A, Reyes-Guerrero DE, Ramírez-Vargas G, Ariza-Román AE, López-Arellano ME, et al. Detection of gastrointestinal nematode populations resistant to albendazole and ivermectin in sheep. Animals. 2019;9:775. 10.3390/ani9100775. 31658591 10.3390/ani9100775 PMC6826479

[93] Qamar W, Alkheraije KA. Anthelmintic resistance in *Haemonchus contortus* of sheep and goats from Asia–A review of *in vitro* and *in vivo* studies. Pak Vet J. 2023;43:376–7. 10.29261/pakvetj/2023.088

[94] Trailović SM, Marjanović ĐM, Trailović JN, Robertson AP, Martin RJ. Interaction of carvacrol with the *Ascaris suum* nicotinic acetylcholine receptors and gamma-aminobutyric acid receptors, potential mechanism of antinematodal action. Parasitol Res. 2015;114:3059–68. 10.1007/s00436-015-4508-x. 25944741 10.1007/s00436-015-4508-x PMC4607277

[95] Marjanović ĐM, Zdravković N, Milovanović M, Trailović JN, Robertson AP, Todorović Z, et al. Carvacrol acts as a potent selective antagonist of different types of nicotinic acetylcholine reeptors and enhances the effect of monepantel in the parasitic nematode Ascaris suum. Vet Parasitol. 2020;278: 109031. 10.1016/j.vetpar.2020.109031. 32032866 10.1016/j.vetpar.2020.109031

